# Evaluation of humoral immune response in relation to COVID-19 severity over 1 year post-infection: critical cases higher humoral immune response than mild cases

**DOI:** 10.3389/fimmu.2023.1203803

**Published:** 2023-07-21

**Authors:** Mi-Seon Bang, Choon-Mee Kim, Nam-Hyuk Cho, Jun-Won Seo, Da Young Kim, Na Ra Yun, Dong-Min Kim

**Affiliations:** ^1^ Department of Internal Medicine, College of Medicine, Chosun University, Gwangju, Republic of Korea; ^2^ Premedical Science, College of Medicine, Chosun University, Gwangju, Republic of Korea; ^3^ Department of Microbiology and Immunology, Seoul National University College of Medicine, Seoul, Republic of Korea

**Keywords:** severe acute respiratory syndrome coronavirus 2, COVID-19, antibody response, neutralizing antibody, neutralization potency, 1-year follow-up

## Abstract

**Introduction:**

Coronavirus disease 2019 (COVID-19) is caused by SARS-CoV-2. We investigated the antibody response against SARS-CoV-2 until 1 year after symptom onset.

**Methods:**

We collected 314 serum samples from 97 patients with COVID-19. Antibody responses were tested using an indirect immunofluorescence assay (IFA), enzyme-linked immunosorbent assay (ELISA), and plaque reduction neutralization test (PRNT) to detect specific neutralizing antibodies.

**Results:**

The positivity rates for neutralizing antibodies at a 1:10 titer cutoff were 58.1% at 1 week, 97.8% at 4 weeks, and 78% at 1 year after symptom onset (53.8% in asymptomatic patients and 89.3% in symptomatic patients). The IFA and anti-S1 ELISA IgG results significantly correlated with neutralizing antibody titers. Critical/fatal cases showed significantly higher antibody titers than the asymptomatic or mild-to-moderate illness groups. Nonetheless, the median number of days to the seroconversion of neutralizing antibodies was 10 and 15 in asymptomatic and symptomatic patients, respectively. The asymptomatic group had a significantly higher neutralizing potency index than the mild-to-severe illness groups.

**Conclusions:**

Neutralizing antibodies corresponded to earlier seroconversion but had a shorter presence in the asymptomatic group than in the symptomatic group and were still present 1 year after symptom onset in critical/fatal cases.

## Introduction

Coronavirus disease 2019 (COVID-19), caused by severe acute respiratory syndrome coronavirus 2 (SARS-CoV-2), has spread rapidly worldwide. Neutralizing antibodies are powerful molecules that constitute a protective immune response against viral infections because they can bind to viral particles and block them from entering the host cells (1, 2). Virus-neutralizing antibodies may be correlated with protection against COVID-19. Therefore, data on the kinetics of virus-neutralizing antibody responses are required (3, 4). Although several studies have shown that protection against COVID-19 may be correlated with the development of high titers of neutralizing antibodies (5, 6), the role of antibodies in COVID-19 is controversial owing to reinfection and the occurrence of severe disease despite high antibody titers. Garcia-Beltran et al. explained the efficacy of the humoral immune response against COVID-19 through quantifying it using the neutralization potency index (titers that achieve 50% neutralization [NT_50_]/immunoglobulin [Ig] G) and showed neutralization potency as a survival predictor (7).

Nonetheless, follow-up studies comparing titers of antibodies, including neutralizing antibodies, based on disease severity and the period after symptom onset have rarely been performed. Therefore, we conducted a 1-year follow-up investigation to examine antibody responses, including neutralizing antibodies, in patients with COVID-19. We analyzed the relationship between the antibody response and neutralizing antibody activity as a function of disease severity and period after symptom onset.

## Materials and methods

### Clinical data and specimens

Clinical data and specimens were obtained from unvaccinated patients COVID-19 who were hospitalized or followed at Chosun University Hospital in Gwangju Metropolitan, South Korea. The individuals recruited for this study were patients with SARS-CoV-2 infection between February 2020 and February 2021, prior to the occurrence of the SARS-CoV-2 variant(s). For diagnosis, a molecular method was used, namely, our in-house-designed reverse transcription polymerase chain reaction (RT-PCR) targeting the *N* gene, and a commercial kit (Kogene Biotech Seoul, South Korea) targeting *E* and *RdRp* genes, according to the manufacturer’s protocol. COVID-19 was diagnosed when more than two genes were detected at a C_t_ value <38 or when the SARS-CoV-2 culture showed a positive result. For patients with a body temperature of ≥37.5°C, the time to fever clearance was defined as the period from the initial fever onset until the body temperature decreased to ≤37.3°C and remained below this temperature for at least 48 h without the use of an antipyretic. COVID-19 patients were divided into four groups based on disease severity: asymptomatic patients (n = 24), those who had no symptoms throughout the course of the infection; patients with mild-to-moderate illness (n = 36), those who were symptomatic but did not receive supplemental oxygen or supplemental oxygen via a nasal prong; severely ill patients (n = 14), those who required high-flow oxygen therapy; and critical/fatal cases (n = 23), those who required mechanical ventilation or died. Chest radiography was performed on the day of hospitalization. X-ray scores were obtained by dividing each lung into upper, middle, and lower zones and scoring each zone from 0 to 4 points based on the degree of infiltration. The scores for each lung (with a total of six zones) were summed to yield a total score of 0–24 (8). The study protocol was approved by the Institutional Review Board of Chosun University Hospital (approval no. CHOSUN 2020-11-007-003). Written informed consent was obtained from all the participants or their legal guardians. All methods were performed in accordance with relevant guidelines and regulations.

### Enzyme-linked immunosorbent assay

The IgG antibodies of patients with COVID-19 were detected using an indirect enzyme-linked immunosorbent assay (ELISA) with the SARS-CoV-2 spike S1 domain recombinant protein as the antigen. The plant-derived S1 recombinant protein was donated by BioApp (Pohang, South Korea). After adding 0.2 μg of S1 recombinant protein to each well of the ELISA plate overnight at 4°C, the wells were washed with phosphate-buffered saline containing 0.05% Tween 20 (PBS-T) solution, followed by the addition of 5% skim milk in PBS-T for blocking. The patient’s serum in 1:100 dilution was added three times to each well for reaction with the antigen protein at 37°C for 2 h. After washing, the diluted horseradish peroxidase-conjugated goat anti-human IgG (Thermo Fisher Scientific), as a secondary antibody, was incubated at 37°C for 1 h. After washing, 50 μL of 3,3′-, 5,5′-tetramethylbenzidine substrate solution was added to each well and incubated at 20–25°C for 30 min. Subsequently, 25 μL of 1 N H_2_SO_4_ was added to stop the reaction. Absorbance at 450 nm (A_450_) was then measured. An A_450_ value of mean + 3 standard deviations or more was established as the positive cutoff value using ELISA on clinical samples from 15 patients who underwent health screening. For each experiment, ELISA was performed by adding a positive control, negative control, and internal control with an A_450_ value of 1.

### Indirect immunofluorescence assay

To perform an indirect immunofluorescence assay (IFA), SARS-CoV-2 samples obtained from the Korea Centers for Disease Control and Prevention were used to infect Vero E6 cells. To prepare the SARS-CoV-2 antigen slide, cells infected for 3 days were cultured on Teflon-coated well slides overnight at 37°C in a 5% CO_2_ environment and fixed with 80% acetone the next day. The patient’s serum was diluted using a twofold serial dilution from 1:16 and then reacted with SARS-CoV-2 antigens in a moist chamber for 30 min at 37°C. After washing, slides were incubated with secondary antibodies at a 1:400 dilution (fluorescein isothiocyanate-conjugated anti-human IgG; MP Biomedicals, OH, USA). The slides were examined under a fluorescence microscope (Olympus IX73, magnification: 400×) after dispensing the mounting solution (Vector Laboratories). An IgG antibody titer of ≥1:32 was established as the cut-off value using IFA on clinical samples from 15 individuals who underwent health screening.

### Plaque reduction neutralisation test

For detection of SARS-CoV-2 neutralizing antibodies, 100 μL of fourfold serially diluted test serum was mixed with 100 μL of 100 plaque-forming units of the SARS-CoV-2 isolate BetaCoV/South Korea/KUMC01/2020 and incubated at 4°C for 1 h. The virus:serum mixture (100 μL) was added to Vero E6 cells (Korean Cell Line Bank, KCLB no. 21587), and adsorption was performed at 37°C in an incubator with 5% CO_2_ for 1 h, after which a 1% methyl cellulose overlay prepared in cell culture maintenance medium (Dulbecco’s modified Eagle’s medium, 5% heat-inactivated fetal bovine serum) was applied. At 5 days post-infection, the inverse of the highest dilution of serum providing 50% (PRNT_50_) viral plaque reduction relative to the virus-only infection was reported as the titer. A 1:10 dilution was considered the lowest possible significant titer. All cell culture infection experiments were performed in a biosafety level 3 laboratory at the Health and Environment Research Institute of Gwangju City and Seoul National University.

To quantify the neutralisation potency of humoral immune responses against SARS-CoV-2, we calculated the neutralization potency index (PRNT_50_/IFA IgG) for each patient.

### Statistical analysis

Statistical analyses were performed using GraphPad Prism 8.0.1 (GraphPad Software, San Diego, CA, USA) and SPSS Statistics 26 (IBM Corp., Armonk, NY, USA). Data on 50% viral plaque reduction relative to virus-only infection (PRNT_50_) were analyzed at 50% inhibition concentration (IC_50_) using GraphPad Prism 8.0.1. A nonparametric multivariate analysis of variance (ANOVA) was performed on the indicated figures. Statistical significance was defined as *p*<0.05. All correlations were analyzed using two-tailed Spearman’s tests; the *r* and *p* values are indicated in the figures. The Kaplan–Meier method was used to estimate seroconversion time using SPSS Statistics 26.

## Results

### Characterization of the tested specimens

**Figure 1:**
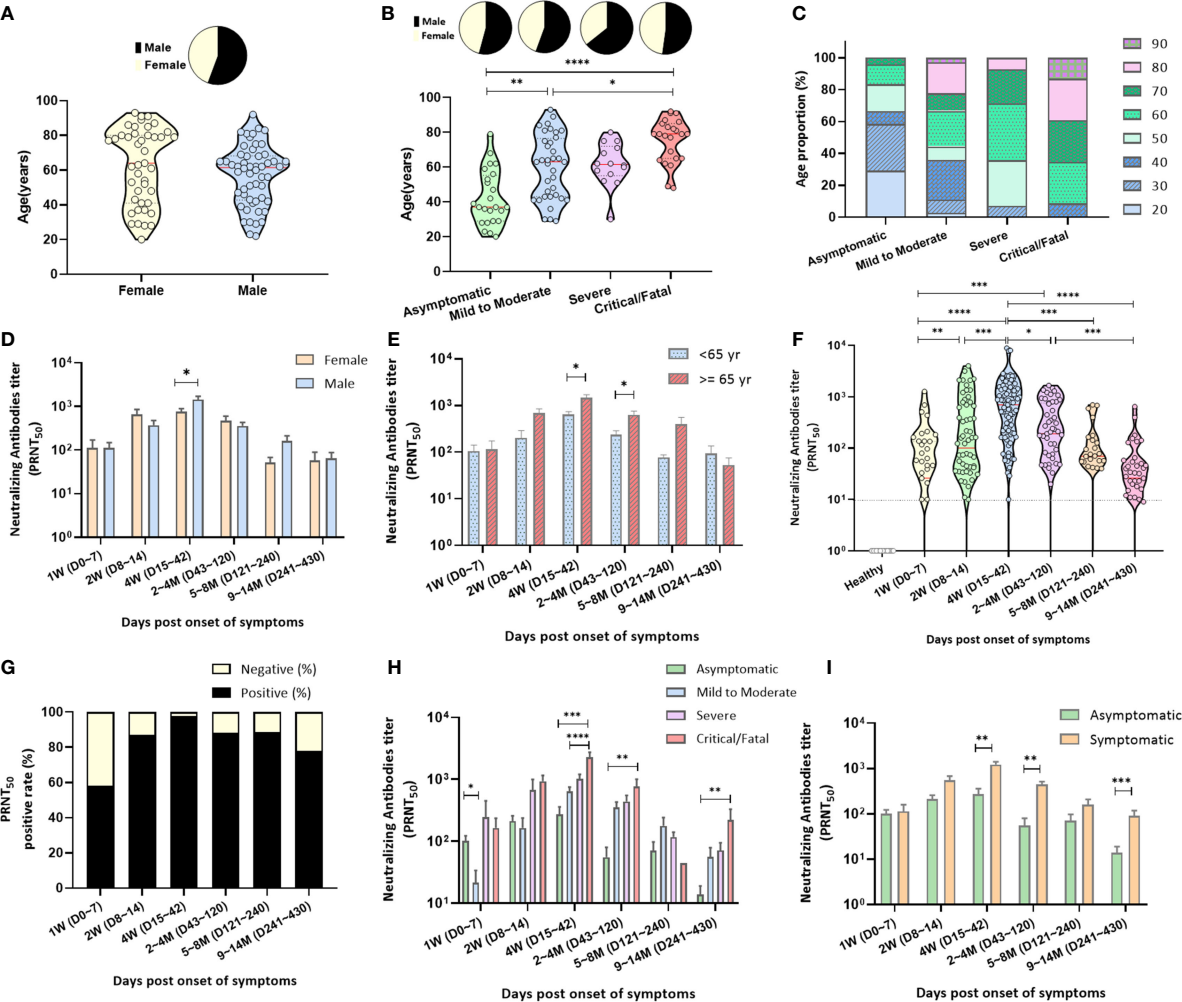
Characterization of the specimens and neutralizing antibody responses. **(A)** Of the enrolled COVID-19 patients, 55.67% were men, with a mean age of 57 (range, 22–92) years, and 44.33% were women, with a mean age of 62.6 (range, 20–93) years. **(B)** COVID-19 patients (n = 97) were subdivided into four groups according to disease severity: asymptomatic cases (n = 24), mild-to-moderate illness (n = 36), severe illness (n = 14), and critical/fatal cases (n = 23). They were analyzed for age and sex by the Kruskal–Wallis test. **(C)** Distribution of age according to disease severity. **(D)** Neutralizing antibodies (PRNT_50_) after symptom onset depending on sex. **(E)** Neutralizing antibodies (PRNT_50_) after symptom onset depending on age. For each parameter, the Mann–Whitney *U* test was performed; statistical significance is indicated as follows: ****p < 0.0001, ***p < 0.001, **p < 0.01, and *p < 0.05. **(F)** Neutralizing antibody titers (PRNT_50_) plotted after symptom onset up to the 1 year of follow-up; negative controls, healthy blood donors (n = 35). The dotted lines indicate the detection limit of 1:10. The red line in the plot denotes the median. **(G)** Relative proportions of cases with undetectable and detectable neutralizing antibodies after symptom onset. Approximately 58%, 87%, 98%, 88%, and 78% COVID-19 patients tested positive for neutralizing antibodies at 0–7, 8–14, 15–42, 43–240, and 241–430 days after symptom onset, respectively. **(H)** Levels of neutralizing antibodies (PRNT_50_) in the four groups after symptom onset **(I)** as well as in asymptomatic and symptomatic patients. Nonparametric ANOVA (Kruskal–Wallis test) was performed; statistical significance is indicated as follows: ****p < 0.0001, ***p < 0.001, **p < 0.01, and *p < 0.05.

Antibody responses in 97 RT-PCR-confirmed individuals with COVID-19 were evaluated at sequential time points. Among the patients, 55.67% and 44.33% were men and women, respectively, with a mean age of 57 (range, 22–92) and 62.6 years (range, 20–93) years, respectively (**Figure 1A**). Patients with critical illness were significantly older (mean age: 75.3, *p* < 0.0001; **Figure 1B**) than those with asymptomatic or mild-to-moderate illness and had a longer duration of fever (mean number of days: 14.61, *p* = 0.0003) than those with asymptomatic-to-severe illness. Baseline characteristics of the disease severity groups are summarized in **Table 1** and **Figure 1**.

**Table 1:** Characterization of enrolled COVID-19 patients according to disease severity on admission.

	Disease severity groups				
	All patients (n=97)	Asymptomatic (n=24)	Mild-to-moderate illness (n=36)	Severe illness (n=14)	Critical/fatal (n=23)
Age (years) Mean ( ± SD) Median (IQR)	59.5 ( ± 19.43) 62 (42.5-77.5)	42.25 ( ± 15.81) 37 (29-55.3)	60.17 ( ± 18.48) 63 (43-76.5)	61.50 ( ± 12.64) 61.5 (55-72)	75.3 ( ± 12.64) 79 (65-85)
Sex Female, n (%) Male, n (%)	43 (44.33%) 54 (55.67%)	9 (47.4%) 10 (52.6%)	14 (43.8%) 18 (56.3%)	5 (35.7%) 9 (64.3%)	10 (47.6%) 11 (52.4%)
Fever duration (days) Mean ( ± SD) Median (IQR)	5.43 ( ± 12.35) 0 (0-4)	0 0 (0)	2 ( ± 6.39) 0 (0-1)	3.1 ( ± 4.73) 1 (0-5.25)	14.61 ( ± 19.01) 8 (0-19.75)
X-ray score Mean ( ± SD) Median (IQR)	4.36 ( ± 4.95) 3 (0-7)	0.55 ( ± 1.81) 0 (0)	1.64 ( ± 2.06) 1 (0-2.25)	8.0 ( ± 5.85) 6.5 (4-11.25)	8.0 ( ± 4.52) 7.5 (4-10.5)
C-reactive protein (mg/dL) Mean ( ± SD) Median (IQR)	6.45 ( ± 7.62) 3.35 (0.4-9.4)	0.45 ( ± 0.55) 0.18 (0.05-0.98)	2.94 ( ± 3.23) 2.15 (0.15-4.9)	4.64 ( ± 3.72) 4.01 (1.4-7.6)	14.62 ( ± 8.47) 19.34 (7.3-21.8)
Lymphocyte count Mean ( ± SD) Median (IQR)	1.85 ( ± 2.93) 1.3 (0.7-2)	2.27 ( ± 1.11) 2.25 (1.7-3)	1.54 ( ± 0.67) 1.57 (0.9-2.1)	0.95 ( ± 0.44) 0.75 (0.6-1.4)	2.49 ( ± 5.75) 0.76 (0.6-0.9)
Persons with other diseases, n (%)	59 (68.6%)	6 (31.6%)	23 (71.9%)	12 (85.7%)	18 (85.7%)

*SD, standard deviation; IQR, interquartile range; dL, deciliter (=10^−4^ m^3^).

### Neutralizing antibody responses

A total of 314 serum samples from 97 patients were tested using the PRNT, and the highest serum dilution that reduced plaque numbers by 50% (PRNT_50_) was determined using the detection limit of the 1:10 antibody titer. The 35 serum samples from healthy subjects contained neutralizing antibodies at a titer <1:10 (**Figure 1F**). Of the 314 serum samples collected from individuals 0 to 430 days after symptom onset, 85.35% tested positive for the PRNT_50_ antibody test.

The neutralizing antibody titer was 111 ± 35.34 (mean ± SD; positivity rate: 58.14%) at 1 week after symptom onset and increased to 500 ± 105.5 (positivity rate: 87.1%) at 2 weeks (**Figures 1F, G**, **Supplementary Table 1**). The neutralization peak occurred at 15–42 days after symptom onset (PRNT_50_ antibody titer: 1120 ± 161.6), where 97.8% of serum samples tested positive in the PRNT_50_ antibody titer test (**Figures 1F, G**, **Supplementary Table 1**).

After 43 days, the number of serum samples with high PRNT_50_ titers decreased significantly. Of the 41 serum samples, 32 (78.05%) collected within 241–430 days after symptom onset remained positive for the PRNT_50_ antibody titer at 62.6 ± 18.36 (mean ± SD; **Figures 1F, G**, **Supplementary Table 1**). Furthermore, men had higher concentrations of neutralizing antibodies in serum than females did (1435 ± 271.6 versus 753.4 ± 131.5, *p* = 0.0343) at 15–42 days after symptom onset (**Figure 1D**, **Supplementary Table 2**). The neutralizing antibody level in patients aged ≥65 years was higher than that in patients aged <65 years, 15–120 days after symptom onset (**Figure 1E**).

The neutralizing antibody response depending on disease severity at different time points after symptom onset was as follows: the PRNT_50_ antibody titers in the critical/fatal cases were significantly higher than those in the mild-to-moderate illness and asymptomatic groups at 15–42 days after symptom onset (**Figure 1H**, **Supplementary Table 4**). At 1-year follow-up, the neutralizing antibody levels in the serum of asymptomatic patients were significantly lower than those of symptomatic patients (*p* = 0.0007; **Figure 1I**, **Supplementary Table 3**), particularly in critical/fatal cases (*p* = 0.0016; **Figure 1H**, **Supplementary Table 4**).

The neutralizing antibody response depending on antiviral drug treatment at different time points after symptom onset was as follow: the PRNT_50_ antibody titers in patients treated with lopinavir/ritonavir or remdesivir were significantly higher than those in patients with non-antiviral treatment 15–42 days after symptom onset (*p* < 0.001; **Supplementary Table 6**). At 1-year follow-up, the neutralizing antibody levels were almost the same in the antiviral treatment and non-treatment groups.

### Antibody responses to SARS-CoV-2

The IgG response against SARS-CoV-2 was measured using IFA and ELISA at multiple time points (**Supplementary Figures 1A, B**). In total, 249 serum samples from 89 patients were tested via IFA using a SARS-CoV-2 antigen slide with a detection limit of a 1:32 antibody titer. The amount of IgG antibodies based on the IFA increased with time after symptom onset, peaking 15–42 days after symptom onset (IFA IgG titer: 864 ± 103.3; **Supplementary Figure 1A**, **Supplementary Table 1**). The IgG antibodies detected by IFA appeared to persist within the analyzed timeframe for up to 1 year. The IFA IgG antibody positivity rates were 41.03% at 1 week, 100% at 4 weeks, and 61.11% at 1 year after symptom onset. A total of 288 serum samples from 95 patients were tested through ELISA for the S1 antigen with an antibody titer detection limit corresponding to A_450_ of 0.3. The amount of anti-S1 ELSIA IgG increased 4 months after symptom onset and showed a tendency to decrease gradually thereafter (**Supplementary Figure 1B**, **Supplementary Table 1**). In contrast, the positivity rate of Anti-S1 ELISA IgG was 90% even at 1 year, unlike the IFA IgG result.

### Antibodies and disease severity

**Figure 2:**
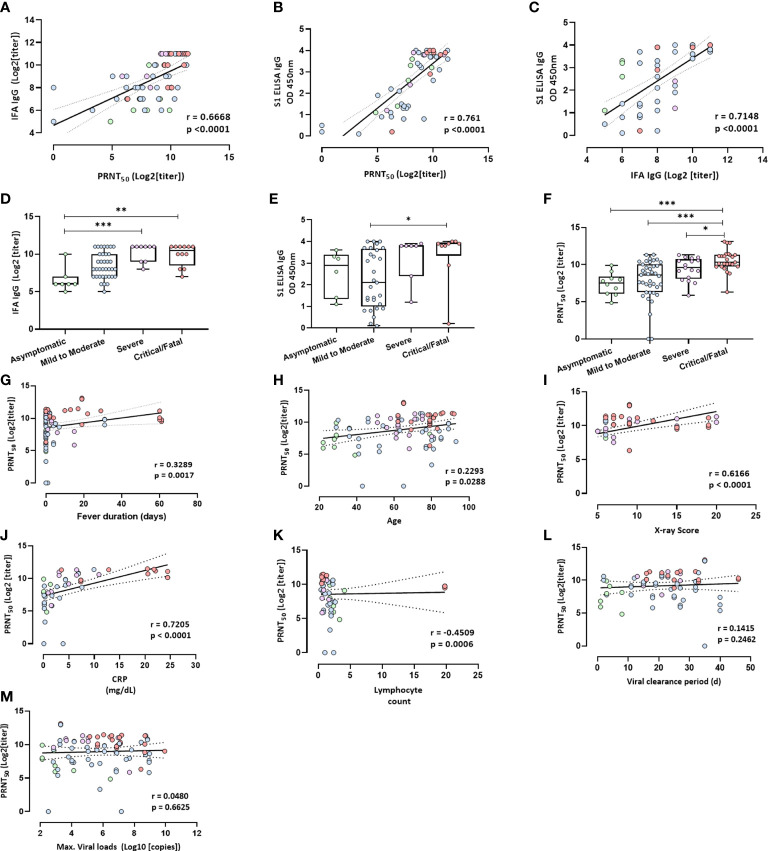
Antibody levels and clinical factors. **(A)** Neutralizing antibody (PRNT_50_) levels plotted against IFA IgG antibody levels at 15–42 days after symptoms onset. *R* = 0.6668, *p* < 0.0001. **(B)** Neutralizing antibody (PRNT_50_) levels plotted against the anti-S1 IgG antibody levels based on an ELISA at 15–42 days after symptom onset; *r* = 0.761, *p* < 0.0001. **(C)** IFA IgG antibody levels plotted against anti-S1 IgG antibody levels at 15–42 days after symptom onset; *r* = 0.7148, *p* < 0.0001. Spearman’s test and linear regression analysis (black line with 95% confidence interval) were performed. **(D)** The IFA IgG antibody titer at 15–42 days after symptom onset. **(E)** Anti-S1 IgG antibody levels based on an ELISA. **(F)** Neutralizing antibody titer (PRNT_50_). Nonparametric ANOVA (Kruskal–Wallis test) was performed; statistical significance is indicated as follows: ***p < 0.001, and **p < 0.01, *p < 0.05. **(G–M)** Clinical factors plotted against levels of neutralizing antibodies (PRNT_50_) at 15–42 days after symptom onset. **(G)** Fever duration; *r* = 0.3289, *p* = 0.0017. **(H)** Age; *r* = 0.2293, *p* = 0.0288. **(I)** X-ray score at the time of hospitalization; *r* = 0.6166, *p* < 0.0001. **(J)** CRP (mg/dL) within 1 week after symptom onset; *r* = 0.7205, *p* < 0.0001. **(K)** Lymphocyte count within 1 week after symptom onset; *r* = −0.4509, *p* = 0.0006. **(L)** Viral clearance period in days; *r* = 0.1415, *p* = 0.2462. **(M)** Maximum viral load in respiratory secretions in the initial infection phase; *r* = 0.048, *p* = 0.6625. Spearman’s test and linear regression analysis (black line with 95% confidence interval) were performed.

The relationships among all individual antibodies measured at the neutralizing antibody peak (15–42 days after symptom onset) and IFA IgG levels correlated with neutralization (*r* = 0.67, *p* < 0.0001; **Figure 2A**). Anti-S1 IgG ELISA also showed a strong correlation with neutralization (*r* = 0.76, *p* < 0.0001; **Figure 2B**). Anti-S1 ELISA IgG levels positively correlated with IFA IgG levels (*r* = 0.72, *p* < 0.0001; **Figure 2C**). The humoral antibody response and neutralizing antibody titers 15–42 days after symptom onset were higher in the critical/fatal cases than in the mild-to-moderate-illness group (**Figures 2D–F**).

### Antibody levels and clinical factors

We analyzed the potential correlation between antibody levels at 15–42 days after symptom onset and several factors, including fever duration, the viral clearance period, maximum viral load, age, an X-ray score, the C-reactive protein (CRP) level (mg/dL), and the lymphocyte count (**Figures 2G–M**). Neutralizing antibody levels were significantly and positively correlated with fever duration, age, the X-ray score at the time of hospitalization, and the CRP level (mg/dL) within 1 week after symptom onset (**Figures 2G–J**) but not with the viral clearance period and maximum viral load in respiratory secretions during the initial infection phase (**Figures 2L, M**). Neutralizing antibody levels were negatively correlated with the lymphocyte count at 1 week after symptom onset (**Figure 2K**). The IgG antibody detected by the IFA was correlated with fever duration, maximum viral load, and age but not with the viral clearance period (**Supplementary Figures 2A–D**). The anti-S1 ELISA IgG antibody was correlated with fever duration but not with the viral clearance period, maximum viral load, and age (**Supplementary Figures 2E–H**).

### Seroconversion

**Figure 3:**
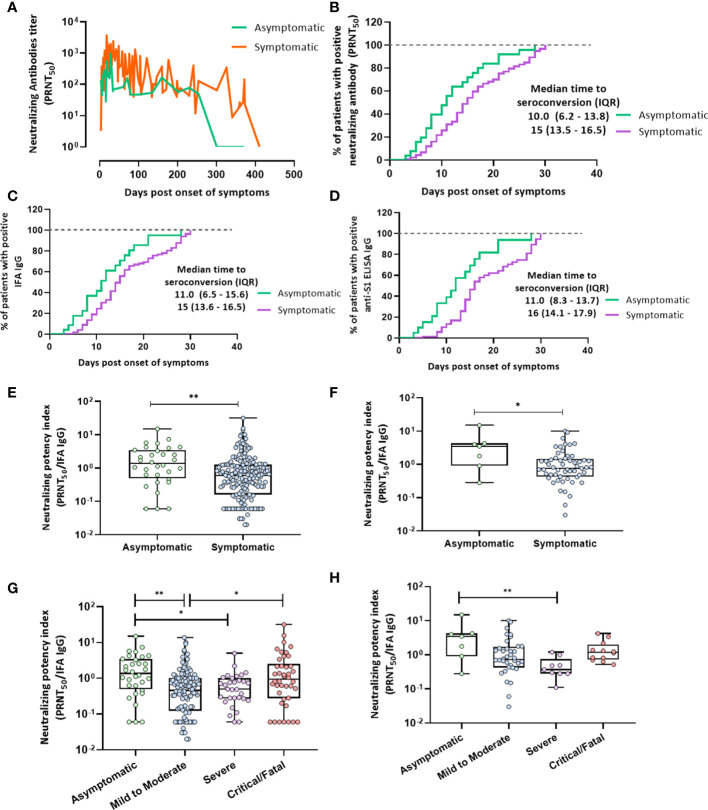
Seroconversion in terms of PRNT_50_, IFA IgG antibodies, and the anti-S1 IgG antibody and the neutralization potency index. **(A)** Neutralizing antibody kinetics during 1-year follow-up in asymptomatic and symptomatic patients as plotted by connecting the line that represents the daily levels of each titer. Mann–Whitney *U* test was performed (*p* = 0.0006). **(B)** Median number of days to seroconversion in terms of neutralizing antibodies within 30 days after symptom onset in asymptomatic or symptomatic patients as analyzed using the Kaplan–Meier method (*p* = 0.002). **(C)** IFA IgG (*p* = 0.004). **(D)** Anti-S1 IgG (*p* = 0.0004). **(E, F)** IFA IgG neutralization potency index (PRNT_50_/IFA IgG) calculated for asymptomatic and symptomatic patients. **(E)** Overall period **(F)** at peak antibody responses. The Mann–Whitney *U* test was performed; statistical significance is indicated as follows: **p < 0.01 and *p < 0.05. **(G, H)** IFA IgG neutralization potency index (PRNT_50_/IFA IgG) in the four groups of disease severity. **(G)** One-year overall follow-up. **(H)** At peak antibody responses. The Kruskal–Wallis test was performed; statistical significance is indicated as follows: **p < 0.01 and *p < 0.05.

Serum samples obtained from 60 patients who achieved seroconversion in terms of neutralizing or other humoral response antibodies were analyzed 30 days after symptom onset (**Figures 3B–D**). The kinetics of neutralizing antibodies at 1-year follow-up in asymptomatic and symptomatic patients are presented in **Figure 3A**. The number of neutralizing antibodies in asymptomatic patients decreased remarkably within one year of symptom onset. The results showed that the time to the emergence of the peak of the neutralizing antibody against SARS-CoV-2 was shorter in asymptomatic patients than in symptomatic patients (*p* = 0.0006; **Figure 3A**). The median number of days to the seroconversion of neutralizing antibodies in asymptomatic patients was 10 days after symptom onset, which was shorter than that in symptomatic patients (15 days, *p* = 0.002; **Figure 3B**). The median number of days to IgG IFA and anti-S1 ELISA IgG seroconversion in asymptomatic patients was 11 days after symptom onset, which was also earlier than in symptomatic patients (15 days, *p* = 0.004 and 16 days, *p* = 0.0004, respectively; **Figures 3C, D**). The median number of days to neutralizing antibody seroconversion was lower in the asymptomatic group than in the mild-to-moderate illness (16 days, *p* = 0.003) and critical/fatal illness groups (15 days, *p* = 0.008; **Figure S3A**). The median number of days for IgG IFA and anti-S1 ELISA IgG antibody seroconversion was similar to that for neutralizing antibody seroconversion (**Supplementary Figures 3B, C**).

### Neutralization potency index

This index was significantly lower in symptomatic than in asymptomatic patients at 1-year follow-up and at the neutralizing antibody peak (**Figures 3E, F**). Furthermore, the neutralization potency index was significantly lower in patients with mild-to-moderate illness and severely ill patients than in asymptomatic patients at 0–430 days after symptom onset (**Figure 3G**). In contrast, the neutralization potency index was higher in critical patients than in those with mild-to-moderate illnesses (**Figure 3G**). This index was significantly lower in severely ill patients at peak neutralizing antibody levels than in asymptomatic patients (**Figure 3H**).

## Discussion

The PRNT is the gold standard assay for assessing neutralizing antibody titers and involves a 50% reduction in plaque number, which is an established endpoint for evaluating serum neutralizing titers (9). In this study, 15–240 days after symptom onset, >88% of serum samples remained antibody positive in the PRNT_50_ assay. Some studies indicate that the neutralizing activity in COVID-19 patients reaches a peak 31–35 days after symptom onset and that at this point, approximately 95% of the patients are test positive based on the detection limit of a 1:160 PRNT_50_ titer (10). In this study, the neutralization peak occurred 15–42 days after symptom onset, and 97.8% of the patients with COVID-19 were positive for the PRNT_50_ titer. Furthermore, 78% of serum samples collected 1 year after symptom onset remained positive for the PRNT_50_ titer at 62.59 ± 18.36.

Antibodies reactive to the receptor-binding domain of SARS-CoV in a pseudoviral system remain detectable for at least 3 years, with 95% of convalescent patients being seropositive at 3 years postinfection (11). Neutralizing antibodies against SARS-CoV during 3-year follow-up were detected in 84% of patients with a 1:10 detection limit, and a 100% positivity rate was noted during the 1-year follow-up (12). Neutralizing antibodies against Middle East respiratory syndrome coronavirus (MERS-CoV) are detectable in 86% of patients with a 1:20 detection limit at 1-year follow-up and remain detectable in these patients for nearly 3 years (13). Our findings showed that neutralizing antibodies against SARS-CoV-2 were present in 78% of COVID-19 patients at the 1-year follow-up at a 1:10 detection limit, 61% at 1:20, and 39% at 1:40. At the 1-year follow-up, the proportion of serum samples with a neutralizing antibody titer ≥1:160 was only 10.71% (3/28) in symptomatic patients, whereas neutralizing antibodies were undetectable in asymptomatic patients. These data indicate that neutralizing antibodies against SARS-CoV-2 did not persist until the end of the 1-year follow-up period, in contrast to antibodies against SARS-CoV or MERS-CoV. Further studies are needed to confirm that the risk of SARS-CoV-2 reinfection is greater than that of SARS-CoV or MERS-CoV reinfection owing to the shorter longevity of SARS-CoV-2-neutralizing antibodies.

In some studies, most patients with severe MERS-CoV infection demonstrated strong long-term antibody responses, whereas some patients with mild infection had very weak or no antibody responses (14). Similarly, patients with severe COVID-19 have higher peak titers of PRNT_50_ and PRNT_90_ antibodies than those with mild or asymptomatic COVID-19 (9, 15, 16). In this study, critical patients with COVID-19 had the highest levels of neutralizing antibodies. In addition, the neutralizing antibody titers in the antiviral drug patients treated with lopinavir/ritonavir or remdesivir were significantly higher than those in the non-antiviral treatment patients 15–35 days after symptom onset. Patients were treated for relatively severe cases; therefore, their PRNT_50_ antibody titers were higher than those in untreated patients.

A 5-month follow-up study of COVID-19 patients has revealed that more than 90% of seroconverters produced detectable neutralizing antibody responses (5). At this time point, these neutralizing antibodies were detectable in 88.5% of the enrolled patients, 80% of the asymptomatic patients, and 90% of the symptomatic patients. In contrast, neutralizing antibodies at 1-year follow-up were present in only 54% (7/13) of asymptomatic patients, 82% (14/17) of patients with mild-to-moderate illness, 100% (4/4) of severely ill patients, and 100% (7/7) of critical/fatal cases, with a titer cutoff of 1:10 (*p* = 0.0016; **Supplementary Table 4**). Furthermore, neutralizing antibodies at a 1:20 titer cutoff were present in 31% (4/13) of asymptomatic patients, 71% (12/17) of patients with mild-to-moderate illness, 100% (4/4) of severely ill patients, and 71% (5/7) of critical/fatal cases (**Supplementary Table 5**). Other studies have suggested that antibody loss in patients with mild COVID-19 is faster than in patients with SARS-CoV infection. They raised concerns that people with mild illness, who comprise the majority of patients with COVID-19, may not achieve sustained humoral immunity against SARS-CoV-2 (12, 17). Our findings show that neutralizing antibodies in asymptomatic patients do not persist until the end of the 1-year follow-up period.

Garcia-Beltran et al. presented a neutralization potency index derived from NT_50_/IgG to assess the quality of anti-RBD IgG antibodies irrespective of the quantity produced. They found that an anti-RBD ELISA IgG neutralization potency index of ≥100 is predictive of 100% 30-day survival and that this neutralization potency is significantly lower in severely ill patients (7). Similarly, our findings revealed that the asymptomatic group had a significantly higher neutralizing potency index than the mild-to-severe group. Our results showed that the neutralization potency index of critical/fatal cases was higher than that of the mild-to-moderate illness group. Conversely, we divided the titers of neutralizing antibodies by IFA IgG titers to calculate the neutralizing potency index and found that the highest values of this index were <10 because the IFA IgG titers varied from negative to 2,048.

Protective correlations that reduce the risk of reinfection by different viruses are generally based on specific antibody levels acquired through vaccination or natural infections (5). In a ferret reinfection model, limited transmission was observed only with a neutralizing antibody titer <1:20, whereas no transmission was observed with this titer ≥1:20 (18). Nonetheless, the levels of neutralizing antibodies that protect against SARS-CoV-2 reinfection in humans remain unknown. Therefore, continuous follow-up must be performed in recovered COVID-19 patients, and monitoring of the lifetime and levels of neutralizing antibodies is necessary.

This study had some limitations. Only a few serum samples were collected from severely and critically ill patients >6 months after symptom onset to determine the antibody kinetics depending on disease severity. In COVID-19 patients, we measured the neutralizing titer against wild-type SARS-CoV-2; however, further research is required to determine whether these serum samples have potential activity against recently emerged SARS-CoV-2 variants (19–21).

In summary, neutralizing antibodies with high potency corresponded to earlier seroconversion, but had a shorter lifetime in asymptomatic patients than in symptomatic patients. Our findings suggest that neutralizing antibody responses to SARS-CoV-2 may not persist for 1 year after symptom onset, particularly in asymptomatic patients, although the levels of these neutralizing antibodies in critical or fatal cases remained high at the 1-year follow-up. In asymptomatic patients, neutralizing antibody responses with high neutralization potency were found but had a short lifetime, with 20% presence at the 1-year follow-up at a neutralizing antibody detection limit of 1:10. Further studies are needed to determine whether reinfection is possible in patients with low NAb levels of neutralizing antibodies.

## Data availability statement

The original contributions presented in the study are included in the article/**Supplementary Material**. Further inquiries can be directed to the corresponding author.

## Ethics statement

The studies involving human participants were reviewed and approved by the Institutional Review Board of Chosun University Hospital (CHOSUN 2020-11-007-003). The patients/participants provided their written informed consent to participate in this study.

## Author contributions

M-SB and C-MK contributed to the investigation and, statistical analyses and drafted the manuscript. D-MK conceived and revised the manuscript. N-HC contributed to the methodology and performed analyses. J-WS, DYK, and NY collected the data and were directly responsible for managing the patients. All authors contributed to the article and approved the submitted version.
