# Supplementary material for: Evaluation of humoral immune response in relation to COVID-19 severity over 1 year post-infection: critical cases higher humoral immune response than mild cases
Source: Front Immunol. 2023 Jul 21;14:1203803. doi: 10.3389/fimmu.2023.1203803 (PMC10401267; doi:10.3389/fimmu.2023.1203803)
Supplement: Supplementary file 1 [file DataSheet_1.docx]

***Supplementary Material***

**Evaluation of humoral immune response in relation to COVID-19 severity over 1 year post-infection: Critical cases higher humoral immune response than mild cases**

Dong-Min Kim^*^, Mi-Seon Bang^†^, Choon-Mee Kim^†^, Nam-Hyuk Cho, Jun-Won Seo, Da Young Kim, Na Ra Yun

*** Correspondence:** Dong-Min Kim: drongkim@chosun.ac.kr

Contents

**Supplementary Figures2**

**Supplementary Figure 1.** Antibody Responses against SARS-CoV-2 in Enrolled Patients after the Onset of Symptoms.2

**Supplementary Figure 2**. Correlation of Antibody Levels with Fever Duration, Viral Clearance, Viral Load, Age, Disease Severity, and Duration of Symptoms during the Infection Period..3

**Supplementary Figure 3**. Seroconversion in Terms of Neutralizing Antibodies (PRNT_50_), IFA IgG, and Anti-S1 IgG in the Four Groups (i.e., Based on Disease Severity) within 30 Days of the Onset of Symptoms.4

**Supplementary Tables5**

**Supplementary Table 1.** Titer of Neutralizing Antibodies (PRNT_50_), IFA Data on IgG, and Anti-S1 IgG (ELISA) Data after Symptom Onset in the Enrolled Patients.5

**Supplementary Table 2.** Neutralizing Antibody Titer (PRNT_50_) by Sex after Symptom Onset..6

**Supplementary Table 3.** Neutralizing Antibody Titer (PRNT_50_) and the Rate of Positivity (%) among Asymptomatic and Symptomatic Patients after Symptom Onset.7

**Supplementary Table 4.** Neutralizing Antibody Titer (PRNT_50_) and the Rate of Positivity (%) in the Four Groups (i.e., by Disease Severity) after Symptom Onset.8

**Supplementary Table 5.** Neutralizing Antibody Positivity Rate (%) in the Four Groups (i.e., by Disease Severity) Approximately 1 Year after Symptom Onset.9

**Supplementary Table 6.** Neutralizing Antibody Titer (PRNT_50_) and Rate of Positivity (%) among Patients with Non-Antiviral and Antiviral treatments after Symptom Onset..10

**Supplementary Figures**

**
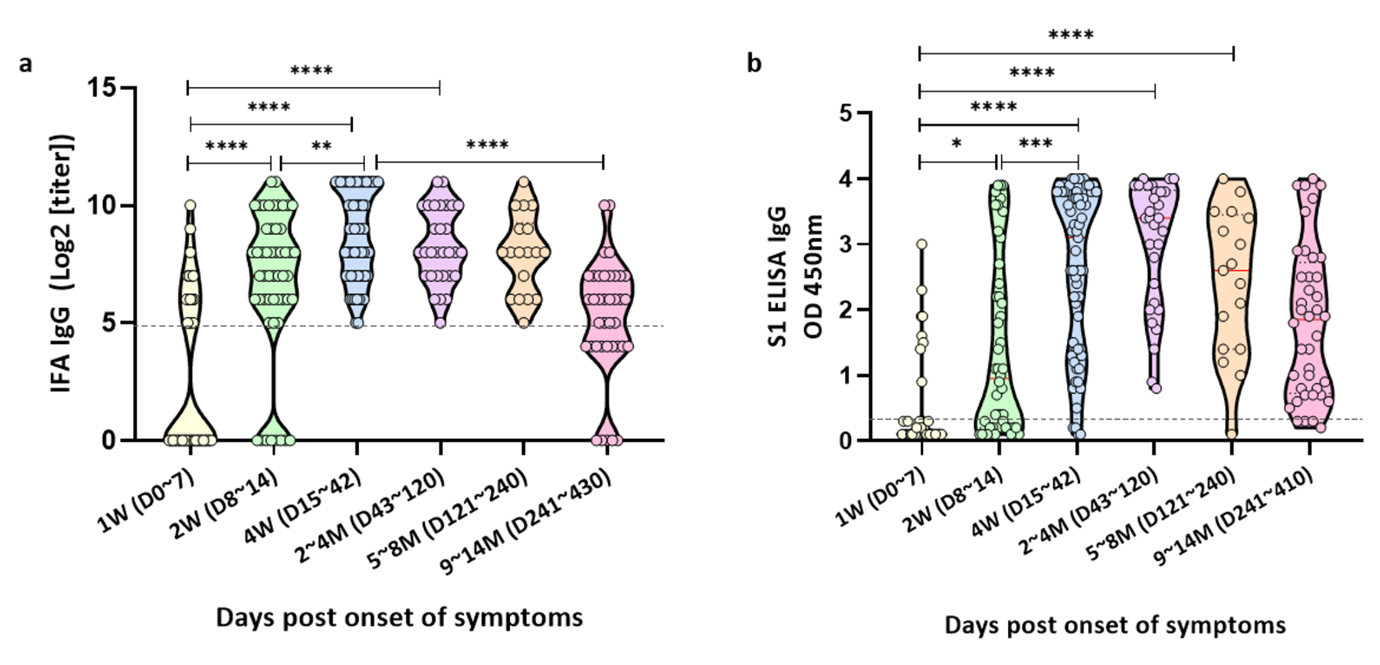
**

**Supplementary Figure 1.** **Antibody Responses against SARS-CoV-2 in Enrolled Patients after the Onset of Symptoms.**

(a) Levels of IFA IgG antibodies after the onset of symptoms are plotted up to 1-year follow-up. (b) The ELISA for the IgG antibody against the S1 antigen. Nonparametric ANOVA (Kruskal-Wallis test) was performed, and statistical significance is presented as follows: *****p* < 0.0001, ****p* < 0.001, ***p* < 0.01, and **p* < 0.05.

**
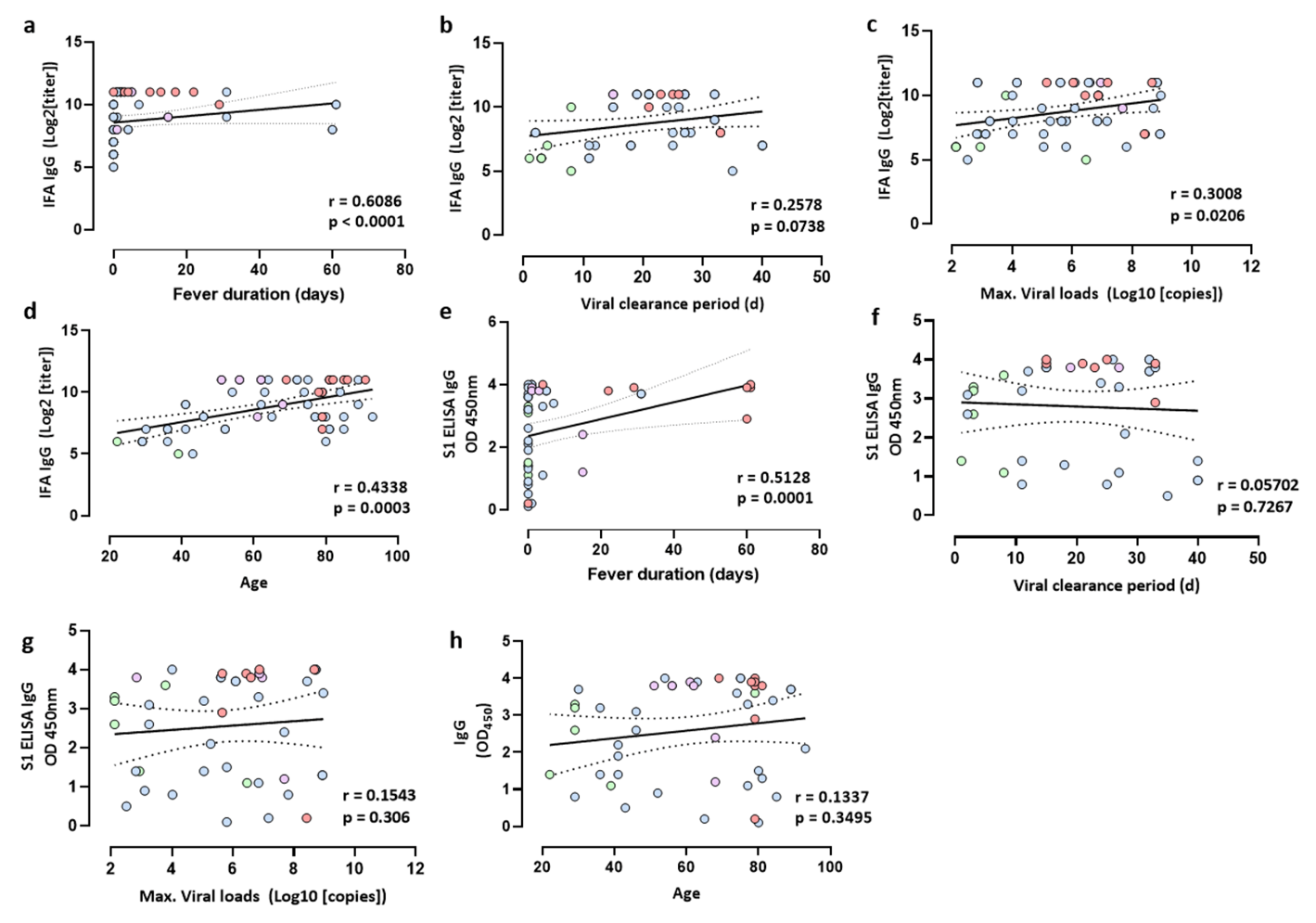
**

**Supplementary Figure 2. Correlation of Antibody Levels with Fever Duration, Viral Clearance, Viral Load, Age, Disease Severity, and Duration of Symptoms during the Infection Period.**

(a–f) Clinical factors plotted against the levels of IFA IgG antibodies at 15–42 days after the onset of symptoms. (a) Fever duration; *r* = 0.6086, *p* < 0.0001. (b) Viral clearance in days; *r* = 0.2578, *p* = 0.0738. (c) Viral load in respiratory secretions during the initial infection phase; *r* = 0.3, *p* = 0.0206. (d) Age; *r* = 0.4338, *p* = 0.0003. (e–h) Clinical factors plotted against anti-S1 IgG antibodies 15–42 days after symptom onset. (e) Fever duration; *r* = 0.5128, *p* = 0.0001. (f) Viral clearance in days; *r* = 0.057, *p* = 0.7267. (g) Viral load in the respiratory secretions during the initial infection phase; *r* = 0.1543, *p* = 0.306. (h) Age; *r* = 0.1337, *p* = 0.3495. Spearman’s test and linear regression analysis (black line with a 95% confidence interval) were performed.

**
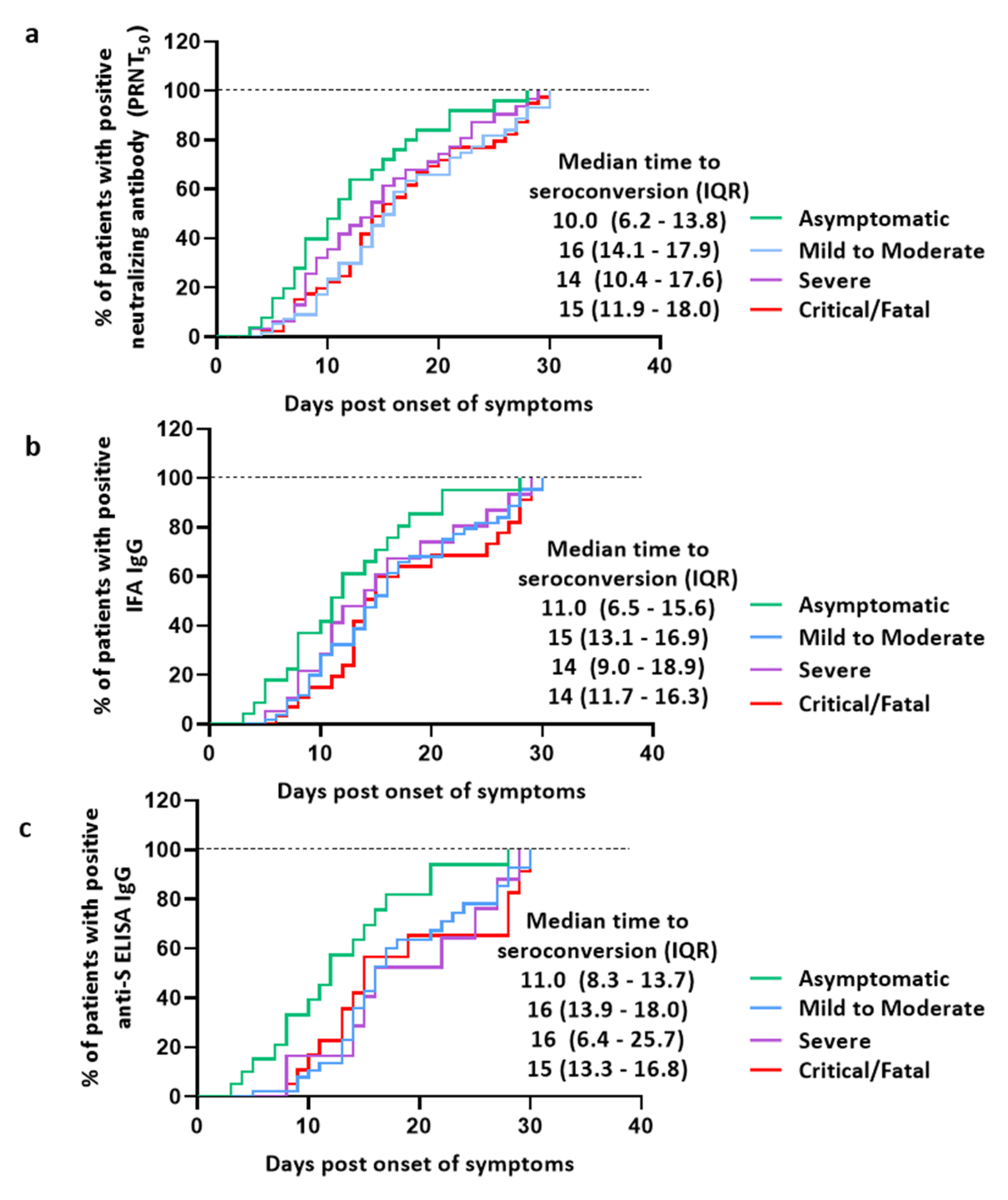
**

**Supplementary Figure 3. Seroconversion in Terms of Neutralizing Antibodies (PRNT_50_), IFA IgG, and Anti-S1 IgG in the Four Groups (i.e., According to Disease Severity) within 30 Days of the Onset of Symptoms.**

(a) Median number of days to neutralizing antibody seroconversion is plotted within 30 days of the onset of symptoms in the four groups using the Kaplan–Meier method (asymptomatic cases vs mild-to-moderate illness: *p* = 0.003, asymptomatic cases vs severe illness: *p* = 0.104, asymptomatic cases vs critical/fatal cases: *p* = 0.008). (b) IFA of IgG antibodies (asymptomatic vs mild-to-moderate: *p* = 0.011, asymptomatic vs severe: *p* = 0.114, asymptomatic vs critical/fatal: *p* = 0.009). (c) Anti-S1 IgG (asymptomatic vs mild-to-moderate: *p* = 0.002, asymptomatic vs severe: *p* = 0.028, asymptomatic vs critical/fatal: *p* = 0.009).

**Supplementary Tables**

**Supplementary Table 1. Titer of Neutralizing Antibodies (PRNT_50_), IFA Data on IgG, and Anti-S1 IgG (ELISA) Data after Symptom Onset in the Enrolled Patients.**

| **Days post onset of symptoms** | **PRNT_50_ antibody titer** | | **IFA IgG antibody titer** | |  | **Anti-S1 ELISA IgG**  **(A_450_)** | |
| --- | --- | --- | --- | --- | --- | --- | --- |
|  | **Mean±SEM** | **Positive (%)** | **Mean±SEM** | **Positive (%)** |  | **Mean±SEM** | **Positive (%)** |
| 1 W (0–7 days) | 111±35.34 | 58.14 | 70.56±29.3 | 41.03 |  | 0.51±0.13 | 22.22 |
| 2 W (8–14 days) | 500±105.5 | 87.10 | 374.8±68.33 | 84.62 |  | 1.42±0.2 | 65.22 |
| 4 W (15–42 days) | **1,120±161.6** | **97.80** | **864±103.3** | **100.00** |  | **2.63±0.16** | **93.44** |
| 2–4 M  (43–120 days) | 399±64.24 | 88.24 | 528±100.5 | 100.00 |  | **3.01±0.18** | **100.00** |
| 5–8 M  (121–240 days) | 151±39.68 | 88.46 | 449.8±123.3 | 100.00 |  | 2.42±0.27 | 94.12 |
| 9–14 M  (241–430 days) | 62.59±18.36 | 78.05 | 109.1±36.11 | 61.11 |  | 1.8±0.2 | 90.00 |

W, weeks; M, months; SEM, standard error of the mean; bold-facing, peak of antibody titer.

**Supplementary Table 2. Neutralizing Antibody Titer (PRNT_50_) by Sex after Symptom Onset.**

| Days post onset of symptoms | **Females** | | | **Males** | | | ***p* value^*^** |
| --- | --- | --- | --- | --- | --- | --- | --- |
|  | n | Mean | SEM | n | Mean | SEM |  |
| 1 W (0–7 days) | 22 | 110.7 | 60.22 | 21 | 110.8 | 37.18 | 0.2506 |
| 2 W (8–14 days) | 28 | 659.9 | 192 | 34 | 368.8 | 107.4 | 0.3962 |
| 4 W (15–42 days) | 42 | 753.4 | 131.5 | 49 | 1435 | 271.6 | **0.0343** |
| 2–4 M (43–120 days) | 18 | 472.3 | 126.8 | 33 | 359.4 | 71.9 | 0.3058 |
| 5–8 M (121–240 days) | 5 | 51.8 | 16.18 | 20 | 163 | 48.82 | 0.2472 |
| 9–14 M (241–430 days) | 12 | 58 | 31.18 | 29 | 64.48 | 22.85 | 0.7819 |

**^*^**For each parameter, the Mann–Whitney *U* test was performed. W, weeks; M, months; SEM, standard error of the mean.

**Supplementary Table 3. Neutralizing Antibody Titer (PRNT_50_) and the Rate of Positivity (%) among Asymptomatic and Symptomatic Patients after Symptom Onset.**

| Days post onset of symptoms | **Asymptomatic cases** | | | **Symptomatic cases** | | | ***p* value^*^** |
| --- | --- | --- | --- | --- | --- | --- | --- |
|  | n | Mean±SEM | Positive (%) | n | Mean±SEM | Positive (%) |  |
| 1 W (0–7 days) | 10 | 100.6±21.81 | 90 | 33 | 113.8±45.76 | 48.48 | **0.0260** |
| 2 W (8–14 days) | 10 | 211.9±46.21 | 100 | 52 | 555.7±124.2 | 84.62 | 0.4812 |
| 4 W (15–42 days) | 8 | 269.6±90.06 | 100 | 81 | 1225±177.8 | 97.53 | **0.0052** |
| 2–4 M (43–120 days) | 6 | 55.5±24.02 | 66.67 | 45 | 445.1±70.01 | 91.11 | **0.0076** |
| 5–8 M (121–240 days) | 5 | 70.6±26.67 | 80 | 20 | 158.3±49.08 | 90.00 | 0.6289 |
| 9–14 M (241–430 days) | 13 | 13.8±5.2 | 53.8 | 26 | 90.5±27.54 | 88.46 | **0.0007** |

**^*^**For each parameter, the Mann–Whitney *U* test was performed. W, weeks; M, months; SEM, standard error of the mean.

**Supplementary Table 4. Neutralizing Antibody Titer (PRNT_50_) and the Rate of Positivity (%) in the Four Groups (i.e., by Disease Severity) after Symptom Onset.**

| Days post onset of symptoms | **Asymptomatic cases** | | | **Mild to moderate illness** | | | **Severe illness** | | | **Critical/fatal cases** | | | ***p* value^*^** |
| --- | --- | --- | --- | --- | --- | --- | --- | --- | --- | --- | --- | --- | --- |
|  | n | Mean±SEM | Positive  (%) | n | Mean±SEM | Positive  (%) | n | Mean±SEM | Positive  (%) | n | Mean±SEM | Positive  (%) |  |
| 1 W (0–7 days) | 10 | 100.6±21.81 | 90 | 15 | 21.33±12.42 | 33.33 | 6 | 243±206.3 | 66.67 | 12 | 164.9±69.76 | 58.33 | **0.0282** |
| 2 W (8–14 days) | 10 | 211.9±46.21 | 100 | 21 | 164.2±73.26 | 80.95 | 13 | 670.5±326.2 | 100 | 18 | 929.6±229.8 | 77.78 | **0.0443** |
| 4 W (15–42 days) | 8 | 269.6±90.06 | 100 | 39 | 639.4±112.4 | 94.87 | 17 | 1021±188 | 100 | 25 | 2278±474 | 100 | **<0.0001** |
| 2–4 M (43–120 days) | 6 | 55.5±24.02 | 66.67 | 27 | 352.1±78.74 | 85.19 | 10 | 441.4±108.5 | 100 | 8 | 763.3±240.9 | 100 | **0.0184** |
| 5–8 M (121–240 days) | 5 | 70.6±26.67 | 80 | 15 | 176.9±64.87 | 86.67 | 4 | 117±23.25 | 100 | 1 | 44±0 | 100 | 0.4609 |
| 9–14 M (241–430 days) | 13 | 13.8±5.2 | 53.8 | 17 | 56.4±22.5 | 82.35 | 4 | 71.5±23.25 | 100 | 5 | 221±107.8 | 100 | **0.0016** |

**^*^**Nonparametric ANOVA (Kruskal–Wallis test) was performed. W: weeks, M: months, SEM: standard error of the mean.

**Supplementary Table 5. Neutralizing Antibody Positivity Rate (%) in the Four Groups (i.e., by Disease Severity) Approximately 1 Year after Symptom Onset.**

| Detection limit  (PRNT_50_ titer) | **All patients** | **Asymptomatic** | **All**  **symptomatic** | **Symptomatic cases** | | |
| --- | --- | --- | --- | --- | --- | --- |
|  |  |  |  | **Mild to moderate** | **Severe** | **Critical/fatal** |
|  | **Positive (%)** | | | | | |
| 1:10 | 78.05 | 53.85 | 89.29 | 82.35 | 100 | 100 |
| 1:20 | 60.98 | 30.77 | 71.43 | 70.59 | 100 | 71.43 |
| 1:40 | 39.02 | 15.38 | 50 | 41.18 | 75 | 57.14 |
| 1:80 | 17.07 | 0 | 25 | 11.76 | 25 | 57.14 |
| 1:160 | 7.32 | 0 | 10.71 | 11.76 | 25 | 14.29 |

**Supplementary Table 6. Neutralizing Antibody Titer (PRNT_50_) and Rate of Positivity (%) among Patients with Non-Antiviral and Antiviral treatments after Symptom Onset.**

| Days post onset of symptoms | **Non-Antiviral treatment** | | | **Antiviral treatment**  **(lopinavir/ritonavir or remdesivir)** | | | ***p* value^*^** |
| --- | --- | --- | --- | --- | --- | --- | --- |
|  | n | Mean±SEM | Positive (%) | n | Mean±SEM | Positive (%) |  |
| 4 W (15–42 days) | 34 | 392±71.2 | 97.1 | 55 | 1598±243.7 | 98.2 | **<0.001** |
| 9–14 M (241–430 days) | 24 | 42.3±16.5 | 75 | 14 | 94.9±45.2 | 78.6 | 0.32 |

**^*^**For each parameter, the Mann–Whitney *U* test was performed. W, weeks; M, months; SEM, standard error of the mean.
